# N6-methyladenosine links RNA metabolism to cancer progression

**DOI:** 10.1038/s41419-017-0129-x

**Published:** 2018-01-26

**Authors:** Dongjun Dai, Hanying Wang, Liyuan Zhu, Hongchuan Jin, Xian Wang

**Affiliations:** 1 Department of Medical Oncology, Sir Run Run Shaw Hospital, Medical School of Zhejiang University, Hangzhou, China; 2 Laboratory of Cancer Biology, Key Lab of Biotherapy, Sir Run Run Shaw Hospital, Medical School of Zhejiang University, Hangzhou, China

## Abstract

N6-methyladenosine (m6A) is the most abundant mRNA modification. With the development of antibody-based sequencing technologies and the findings of m6A-related “writers”, “erasers”, and “readers”, the relationships between m6A and mRNA metabolism are emerging. The m6A modification influences almost every step of RNA metabolism that comprises mRNA processing, mRNA exporting from nucleus to cytoplasm, mRNA translation, mRNA decay, and the biogenesis of long-non-coding RNA (lncRNA) and microRNA (miRNA). Recently, more and more studies have found m6A is associated with cancer, contributing to the self-renewal of cancer stem cell, promotion of cancer cell proliferation, and resistance to radiotherapy or chemotherapy. Inhibitors of m6A-related factors have been explored, and some of them were identified to inhibit cancer progression, indicating that m6A could be a target for cancer therapy. In this review, we are trying to summarize the regulation and function of m6A in human carcinogenesis.

## Facts

- N6-methyladenosine influences almost every step of RNA metabolism;
- N6-methyladenosine plays important roles in cancer progression;
- Chemicals targeting N6-methyladenosine might be a new method of cancer therapy.

## Open questions

- Are there more “writers”, “erasers”, and “readers” in the regulation of N6-methyladenosine? Are they having additional functions?
- Could N6-methyladenosine be an effective target for cancer therapy?
- What is the potential connection of other RNA modifications with N6-methyladenosine? Could they be likewise reversible?

## Introduction

N6-methyladenosine (m6A) is the most abundant messenger RNA (mRNA) modification in mammals. It is now being pushed to the front of the biology science for the discovery of its “writers”, “erasers”, and “readers” that can add, remove, or preferentially bind to the m6A site and alter important biological functions^1^. m6A in isolated RNA is estimated to be 0.1–0.4% in adenines (3–5 m6A sites per mRNA)^2,3^. The m6A occurs mostly in DRACH sequence (where D denotes A/G/U, R denotes A/G, and H denotes A/C/U), which is the m6A consensus motif^4–6^. The m6A is enriched around stop codons, in 3ʹ untranslated regions (3ʹ UTRs) and within internal long exons, and m6A occurs more in precursor mRNAs (pre-mRNAs)^7,8^.

m6A is involved in various aspects of mRNA metabolism including mRNA translation and mRNA decay^9^. Accumulating evidences support the importance of RNA biology in the hallmarks of cancer^10–16^. However, the associations between RNA modification and cancers are rarely reviewed. While there are increasing evidences showing m6A plays diverse roles in cancer development and progression^17–24^, we try to overview the regulation and function of RNA m6A in the process of cancer progression.

## How m6A is regulated

**Fig. 1:**
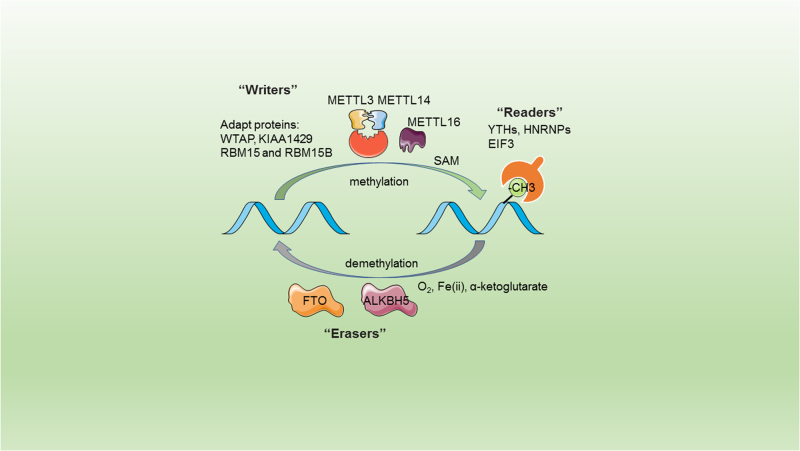
m6A regulation by m6A “writers”, “erasers”, and “readers” m6A modification is conducted by its “writers”, “erasers”, and “readers” to add, remove, or preferentially bind to m6A. The metyltransferase-like 3 (METTL3) and METTL14 form a stable complex with a stoichiometric ratio of 1:1, METTL14 helps METTL3 for substrate recognition. Adapt proteins such as Wilms tumor 1-assocated protein (WTAP), KIAA1429, RNA-binding motif protein 15 (RBM15), and its paralogue RBM15B lead the METTL3–METTL14 complex to certain mRNAs; Fat mass and obesity-associated (FTO) and AlkB homologue 5 (ALKBH5) use O2, Fe(ii), α-ketoglutarate as substrates to demethylate the m6A site. YT521-B homology (YTH) domain-containing protein, eukaryotic initiation factor 3 (EIF3), and the heterogeneous nuclear ribonucleoprotein (HNRNP) protein families recognize the m6A site and bind to it, and function differently in RNA metabolism

m6A is catalyzed by a RNA methyltransferase complex (Fig. 1; Table 1). Methyltransferase-like 3 (METTL3) was identified as the first *S*-adenosylmethionine (SAM)-binding subunit of the RNA methyltransferase complex^25^. METTL3 and methyltransferase-like 14 (METTL14) colocalize in nuclear speckles and form a stable complex with a stoichiometric ratio of 1:1^26^. METTL3 was the active site to bind to SAM while METTL14 plays a structural role critical for substrate recognition^27^. Occasionally, the METTL3–METTL14 heterodimer needs an adaptor protein. Wilms tumor 1-associated protein (WTAP) is the first adaptor identified to interact with both METTL3 and METTL14^26^. Additionally, WTAP interacts with many proteins and long-non-coding RNA (lncRNAs)^28^, indicating that WTAP may recruit other factors to methyltransferase complex. Moreover, other adaptor proteins such as KIAA1429^29^, RNA-binding motif protein 15 (RBM15), and its paralogue RBM15B^30^ were found to be interacted with METTL3 complex and depletion of these adaptors also decreased the cellular m6A level. METTL16 is a newly found m6A methyltransferase, which methylates m6A sites mainly in 3ʹ UTRs, and knockdown of METTL16 led to a ~20% decrease of m6A^31^. Fat mass and obesity-associated (FTO) and AlkB homolog 5 (ALKBH5) are the only two identified m6A demethylases. They are Fe(ii) and α-ketoglutarate dependent, employing ferrous iron as co-factor and α-ketoglutarate as co-substrate to oxidize the *N*-methyl group of m6A site to a hydroxymethyl group^32^. Either deficiency or overexpression of FTO^33^ or ALKBH5^34^ altered the m6A level in cells.

**Table 1:** List of m6A regulators and their roles in RNA metabolism

Type	Names	Functional classification	Functions in m6A regulation and RNA metabolism	References (Pubmed ID)
m6A writer	METTL3	Catalytic m6A methyltransferase	Create m6A sites (most near 3ʹ UTRs, catalytic site is aa 395–398, DPPW), promote mRNA translation (independent of its catalytic function)	24316715
	METTL14	Subunit of METTL3 m6A methyltransferase complex	Help METTL3 to recognize substrate	27373337
	WTAP	Subunit of METTL3 m6A methyltransferase complex	Adaptor protein to lead METTL3–METTL14 heterodimer to mRNAs	24981863
	KIAA1429	Subunit of METTL3 m6A methyltransferase complex	Adaptor protein to lead METTL3–METTL14 heterodimer to mRNAs	24981863
	RBM15 and its paralogue RBM15B	Subunit of METTL3 m6A methyltransferase complex	Determine which DRACH sites are methylated	27602518
	METTL16	Catalytic m6A methyltransferase	m6A sites creation (most in introns, catalytic site is PP185/186 and F187), mRNA splicing, regulation of *S*-adenosylmethionine homeostasis	28525753
m6A eraser	FTO	m6A demethylase (catalytic site is H231 and D233)	RNA demethylation, mRNA splicing	25412662
	ALKBH5	m6A demethylase (catalytic site is H204 or H266)	RNA demethylation, mRNA processing, mRNA exporting, pre-mRNA stability in nuclear speckles	23177736, 28344040
m6A reader	YTHDC1	Direct reader	mRNA splicing	26876937
	YTHDF1	Direct reader	mRNA translation	26046440
	YTHDF2	Direct reader	mRNA decay	27558897
	YTHDF3	Direct reader	Interacted with YTHDF1 and YTHDF2 to facilitate mRNA translation and mRNA degradation	28106072
	HNRNPA2B1	Direct reader	miRNA splicing	26321680
	EIF3	Direct reader	Promote mRNA translation	26046440
	HNRNPC	Indirect m6A reader	mRNA splicing	25719671

*m6A* N6-methyladenosine, *METTL3* metyltransferase-like 3, *METTL14* metyltransferase-like 14, *WTAP* Wilms tumor 1-assocated protein, *RBM15* RNA-binding motif protein 15, *DRACH*, D denotes A/G/U, R denotes A/G, and H denotes A/C/U, which is the m6A consensus motif, *METTL16* metyltransferase-like 16, *FTO* fat mass and obesity-associated, *ALKBH5* AlkB homologue 5, *YTHDC1* YTH domain-containing 1, *YTHDF1* YTH m6A-binding protein 1, *YTHDF2* YTH m6A-binding protein 2, *YTHDF3* YTH m6A-binding protein 3, *HNRNPA2B1* heterogeneous nuclear ribonucleoprotein A2/B1, *miRNA* microRNA, *EIF3* eukaryotic initiation factor 3, *HNRNPC* heterogeneous nuclear ribonucleoprotein C

Similar to DNA methylation, the biological function of m6A is mediated through the recognition of m6A site by m6A “readers”^1,35^. m6A “readers” bind to RNAs by two different patterns, direct reading or indirect reading. Direct reading refers to selective binding of m6A “readers” to m6A site of RNAs. Indirect reading means that m6A modification alters RNA secondary structures and thereby renders the RNA accessible to RNA-binding proteins (termed as “m6A switch”). YTH (YT521-B homology) family proteins YTHDF1-3 and nuclear member YTHDC1 could directly bind to m6A containing RNA. Heterogeneous nuclear ribonucleoprotein A2/B1 (HNRNPA2B1) and heterogeneous nuclear ribonucleoprotein C (HNRNPC) are two abundant nuclear RNA-binding proteins responsible for pre-mRNA processing^36^. m6A site of pre-mRNA indirectly alters the binding of HNRNPC to its U-tract motifs^37^. HNRNPA2B1 directly binds to m6A site of RNA and was identified to be a regulator in microRNA (miRNA) processing^38^. Eukaryotic initiation factor 3 (EIF3) was identified as a direct m6A-binding protein to promote cap-independent translation^39^.

## The biological function of m6A in mRNA metabolism

**Fig. 2:**
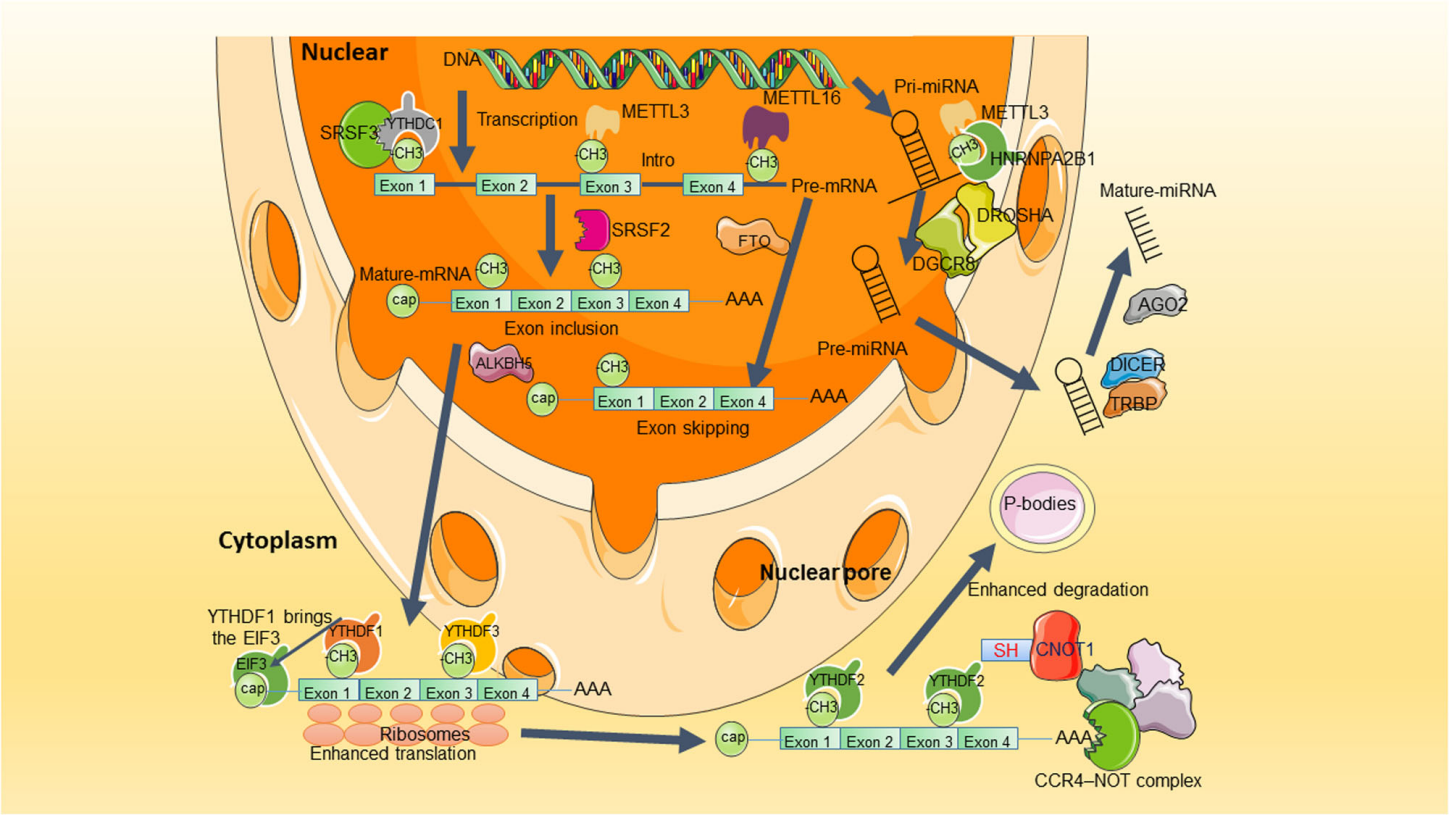
m6A plays important roles in RNA metabolism m6A participates in almost every step in RNA metabolism, after transcription, methyltransferase-like 3 (METTL3) or METTL16 methylate the pre-mRNA, while splicing factor splicing factor, arginine/serine-rich 2 (SRSF2) would be recruited to promote the exon inclusion, in the contrary, if fat mass and obesity-associated (FTO) demethylates the m6A site, there would be an exon skipping. m6A “reader” YTH domain-containing 1 (YTHDC1) binds to m6A site and brings SRSF3 to splice RNA. m6A “reader” heterogeneous nuclear ribonucleoprotein A2/B1 (HNRNPA2B1) induces the recognition of DGCR8 (microprocessor complex subunit) to primary microRNAs (pri-miRNA) and stimulates pri-miRNA processing. AlkB homologue 5 (ALKBH5) can promote the exporting of mRNA from nuclear to cytoplasm. In cytoplasm, YTH m6A-binding protein 1 (YTHDF1) and YTH m6A-binding protein 3 (YTHDF3) would enhance the translation of m6A-modified mRNA. And the m6A-modified mRNA can be recognized by YTH m6A-binding protein 2 (YTHDF2), which binds to SH domain of CCR4–NOT transcription complex subunit 1 (CNOT1). The carbon catabolite repression 4–negative on TATA-less (CCR4–NOT) complex would induce the deadenylation of mRNA and process the decay of mRNA in processing bodies (P-bodies)

RNA metabolism comprises the entire mRNA life from birth to death that includes RNA processing, RNA transporting from nucleus to cytoplasm, RNA translation, and RNA decay. As shown in Fig. 2, the m6A modification affects many aspects of RNA metabolism. RNA processing promotes pre-mRNA to become mature mRNA through three steps, namely 5ʹ capping, 3ʹ polyadenylation, and splicing. m6A was found to be more abundant in pre-mRNA than in mature mRNA^40^. More m6A were found in introns^7,41^. Many m6A “writers”, “erasers”, and “readers” localize predominantly in nuclear speckles^33,34,41,42^, the sub-nuclear structures enriched with pre-mRNA splicing factors. The splicing factor serine and arginine-rich splicing factors (SRSFs) play important roles in mRNA splicing. FTO depletion or METTL3 overexpression increased m6A levels and subsequently promoted SRSF2 binding to facilitate the inclusion of target exons^43^. m6A “reader” nuclear YTH family member YTHDC1 could function as a recruiter to bring SRSF3 to its mRNA-binding regions near m6A. In contrast, SRSF10 might bind to its target mRNAs regions and modulate exon skipping in the absence of m6A modification or YTHDC1^42^. Splicing regulators and m6A “reader” HNRNPC could affect the abundance as well as alternative splicing of target mRNAs in an “m6A switch” regulated manner^38^. And HNRNPA2B1 could regulate alternative splicing of miRNA^38^. U6 small nuclear RNA (snRNA), a component of U6 small nuclear ribonucleoprotein (snRNP) that functions in mRNA splicing, was found to be methylated by METTL16^44^. METTL16 was found to methylate the first hairpin of the six hairpin structures in the 3ʹ UTR of methionine adenosyltransferase 2A (MAT2A) and led to splicing of retained introns to produce more mRNA of MAT2A^31^.

Nuclear export of mRNAs is a crucial step in the regulation of gene expression. m6A was found to promote RNA export from nucleus to cytoplasm. METTL3 knockdown delayed mRNA export^45^ while inhibition of ALKBH5 enhanced mRNA export to the cytoplasm^34^. m6A also facilitates mRNA translation. YTHDF1 interacted with EIF3 to promote the rate-limiting step of translation for m6A-modified mRNAs. After knockdown of YTHDF1, the m6A-modified mRNAs would be less associated with polysomes^46^. After knockdown of YTHDF3 in HeLa cells, the ratio of ribosome-bound fragments and input RNA was downregulated^47^. Further knockdown of METTL3 also downregulated this ratio of the YTHDF3 bounded mRNA targets, suggesting that YTHDF3 promotes mRNA translation in an m6A-dependent manner. In addition, METTL3 can recruit EIF3 to interact with translation initiation machinery and promoted translation of m6A-modified mRNAs independent of its methyltransferase activity^23^. Furthermore, under cellular stresses, m6A occurred at 5ʹ UTR facilitated cap-independent translation, mediating stress-induced translational responses^39^.

RNA decay refers to the degradation of mRNA in decay sites, such as processing bodies. There are different degradation pathways including deadenylation-dependent decay pathway, which starts with shortening of polyadenylation tail by deadenylases such as carbon catabolite repression 4–negative on TATA-less (CCR4–NOT) complex. m6A modification was found to promote deadenylation of RNAs. YTHDF2 directly interacted with the SH domain of CCR4–NOT transcription complex subunit 1 (CNOT1), a scaffolding subunit of CCR4–NOT complex, mediating RNA deadenylation and RNA degradations^48^. YTHDF2 knockdown prolonged lifetimes of its mRNA targets. After YTHDF2 knockdown, a 21% increase of the m6A/A ratio of the total mRNA was observed^49^. Furthermore, YTHDF1 and YTHDF3 can potentially affect the partitioning of methylated transcripts to YTHDF2 for decay. Inhibition of METTL3 or METTL14 had also been shown to increase the expression of target mRNAs^26,49^.

As shown above, m6A “writers” give methyl group to RNAs and different “readers” recognize those m6A-modified RNAs for different functions. m6A-modified RNAs experience a faster journal for RNA processing, export, translation, and decay. This fast-tracking model allows cells to generate enough proteins to cope with different situations. For example, EIF3 could recognize m6A-modified RNAs and promoted the cap-independent translation under stress condition^39^, which is associated with cancer progression^50^.

## m6A in the regulation of non-coding RNAs

Besides mRNA, non-coding RNAs are also regulated by m6A^51^. LncRNA and miRNA are two major classes of non-coding RNAs. LncRNA plays important parts in chromatin organization, transcriptional, and post-transcriptional regulation^52,53^. HNRNPC was found to bind 2577-m6A hairpin compared to the unmethylated hairpin of the lncRNA metastasis-associated lung adenocarcinoma transcript (MALAT1) in an “m6A switch”-regulated manner, which indicated m6A modification acted as a trigger to disrupt lncRNA structure^38^. Inhibition of MALAT1 suppressed cancer cells proliferation and invasion^54^. Accordingly, the alteration of MALAT1 splicing by m6A “reader” might associate with cancer progression. LncRNA X-inactive-specific transcript (XIST) mediated the transcriptional silencing of genes on the X chromosome. RBM15 and RBM15B recruited METTL3 to methylate XIST. Knockdown of RBM15 and RBM15B, or METTL3 was shown to impair XIST-mediated gene silencing both in intro and in vivo^30^.

miRNA could target specific mRNA sites and promote degradation or translation inhibition of mRNA^14^. m6A sites and miRNAs-targeted sites are sometime overlapped at the 5ʹ end and 3ʹ end of 3ʹ UTRs^55^. Primary miRNA (pri-miRNA) transcript is cleaved in the nucleus by Drosha and DGCR8, (microprocessor complex subunit), forming the precursor miRNA (pre-miRNA)^14^. METTL3 methylated pri-miRNAs^56^, marking them for recognition and processing by DGCR8. Consistently, METTL3 depletion reduced the binding of DGCR8 to pri-miRNAs and led to reduction of mature miRNAs and accumulation of unprocessed pri-miRNAs. METTL3 and METTL14 methylated developmental-related RNA and m6A methylation blocked ELAV-like RNA-binding protein 1 (HUR) binding^49^, resulting in transcript destabilization. Knockdown of METTL3 or METTL14 reduced m6A to increase HuR–mRNA interaction and prevent miRNA binding.

## m6A and stem cell differentiation

By mapping the m6A methylome in embryonic stem cells, thousands of mRNAs and lncRNAs including transcripts encoding core pluripotency transcription factors, were found to show conserved m6A modification^57^. Knockdown of METTL3 or METTL14 in mouse embryonic stem cells (mESC) led to loss of m6A and self-renewal capability, with most pluripotent factors being downregulated while some developmental regulators significantly upregulated^49^. In contrast, m6A seems to be required for embryonic stem cells to rapidly exit the pluripotent state upon differentiation^57^. In fact, mESC with METTL3 depletion renewed at an improved rate. Zinc-finger protein 217 (ZFP217) interacted with METTL3 to inhibit m6A deposition on transcripts of pluripotency genes^58^. ZFP217 depletion impaired ESC self-renewal and somatic cell reprogramming by increasing m6A RNA levels and promoting degradation of m6A-modified mRNAs of pluripotency factors such as homeobox transcription factor Nanog (NANOG), transcripts POU domain, class 5, transcription factor 1 (POU5F1), Krueppel-like factor 4 (KLF4), SRY-box 2 (SOX2), and MYC proto-oncogene (C-MYC). This discrepancy could potentially be explained by different dependencies of pluripotent factors and developmental regulators on m6A modification. The influence of m6A upon cell fate transition in stem cells also seems to exist in cancer stem cells (CSC)^22,24^.

## The biological function of m6A in cancer progression

Cancer has many potential links with m6A modifications. For example, alternative pre-mRNA splicing often presents in cancer, which was found to be regulated by m6A^59,60^. After silencing of METTL3, a noteworthy enrichment of the P53 signaling pathway was found, and gene expression and alternative splicing patterns related to this pathway were changed^51^. HUR is highly expressed in many cancers and is found as an RNA stabilizer protein^61–64^. Two hallmarks of m6A were concluded that it serves as a marker to group and synchronize cohorts of transcripts for fast-tracking mRNA processing and metabolism; and that it considerably affects cell-state transition during cell differentiation^9^. Both hallmarks are related to cancer progression. Besides, m6A is considered to influence miRNA processing^55^ and lncRNA splicing^38^ that might alter cancer progression^65,66^. And next we will review the relationships between m6A and cancer in line with different cancer types (Table 2).

**Table 2:** m6A regulators are associated with cancer

m6A regulator	Cancer type	Details	References (Pubmed ID)
METTL3	Lung cancer	METTL3 promotes growth, survival, and invasion of human lung cancer cells by increasing translation of mRNAs	27117702
METTL14	Hepatocellular carcinoma	By interacting with DGCR8, METTL14 increases primary miRNA 126 and decreases mature miRNA 126 in an m6A-dependent manner, while mature miRNA 126 inhibits the repressing effect of METTL14 in tumor metastasis	27774652
FTO, METTL3	Osteosarcoma, malignant melanoma	m6A RNA serves as a beacon for the selective, rapid recruitment of Pol κ to damage sites to facilitate repair and cell survival	28297716
FTO, METTL3, or METTL14	Glioblastoma	m6A RNA methylation regulates the self-renewal and tumorigenesis of glioblastoma stem cells by regulating mRNA m6A enrichment and expression. FTO inhibitor MA2 suppresses glioblastoma progression at in vitro and in vivo studies	28297667
FTO	Acute myeloid leukemia	High expression of FTO promotes leukemogenesis in subtypes of AML. By lowering the m6A level, FTO downregulates, key targets ASB2 and RARA expression, and inhibits all-trans-retinoic acid-induced leukemia cell differentiation	28017614
ALKBH5	Glioblastoma	High expression of ALKBH5 promotes self-renewal and tumorigenesis of glioblastoma stem-like cells through regulation of FOXM1. The lncRNA antisense to FOXM1 links ALKBH5 to FOXM1 nascent RNA, leading to demethylation and elevated expression of FOXM1	28344040
ALKBH5	Breast cancer	Hypoxia-inducible factors promote pluripotency factor expression of breast cancer stem cell by inducing ZFP217-dependent inhibition of m6A methylation under hypoxic tumor microenvironment	27590511
ALKBH5	Breast cancer	Hypoxia-inducible factors-dependent ALKBH5 expression mediates enrichment of BCSCs by decreasing NANOG mRNA methylation and increasing its mRNA levels	27001847
YTHDF2	Hepatocellular carcinoma	miR 145 modulates m6A levels by targeting the 3ʹ UTR of YTHDF2 mRNA in HCC cells	28104805

*m6A* N6-methyladenosine, *METTL3* metyltransferase-like 3, *METTL14* metyltransferase-like 14, *DGCR8*, *DGCR8*, microprocessor complex subunit, *miRNA* microRNA, *FTO* fat mass and obesity-associated, *Pol κ* DNA polymerase κ, *MA2* the ethyl ester derivative of meclofenamic acid, *AML* acute myeloid leukemia, *ASB2* ankyrin repeat and SOCS box protein 2, *RARA* retinoic acid receptor-a, *ALKBH5* AlkB homologue 5, *lncRNA* long-non-coding RNA, *FOXM1* fork head box M1, *ZFP217* zinc-finger protein 217, *BCSCs* breast cancer stem cells, *NANOG* homeobox transcription factor Nanog, *YTHDF2* YTH m6A-binding protein 2

### m6A and breast cancer

Breast cancer stem cells (BCSCs) is a group of subpopulation cells capable of infinite proliferation through self-renewal. Only BCSCs can form recurrent or metastatic tumor^67,68^. BCSCs phenotype is caused by core pluripotency factors^69–73^. Hypoxia could induce the expression of ALKBH5 and decrease m6A modification in breast cancer cells^24^, in a HIF-dependent manner. NANOG was found to be upregulated because the demethylation stabilizes NANOG mRNA. Depletion of ALKBH5 impaired hypoxia-induced BCSCs enrichment, while ALKBH5 overexpression phenocopied the effect of hypoxia. Hence, HIF-dependent ALKBH5 expression mediated enrichment of BCSCs in hypoxic tumor microenvironment^24^. Similarly, hypoxia induced ZFP217-dependent inhibition of m6A methylation of mRNAs encoding pluripotency factors that mediated BCSCs specification in breast cancer cells^22^. In addition, METTL14 was significantly decreased in breast cancer and lower METTL14 of breast cancer was associated with a shorter survival (RFS) of breast cancer patients^21^.

### METTL3 promotes translation of oncogenes in human lung cancer

Knockdown of METTL3 downregulated epidermal growth factor receptor (EGFR) protein level^23^. Polysome profiling assay found METTL3 enhanced the translation of oncogenes. However, tethering reporter assay showed tethering METTL3 to a luciferase mRNA-enhanced translation independent of its methyltransferase activity. In fact, METTL3 interacted with translation initiation factors such as nuclear cap-binding protein subunit 1 (CBP80) and eukaryotic translation initiation factor 4E (EIF4E) in an RNA-independent manner, and METTL3 specifically promoted translation of initiation factor-dependent reporter mRNAs. By doing so, METTL3 promoted growth, survival, and invasion in lung cancer cells. Another study found that miR-33a attenuated non-small-cell lung cancer (NSCLC) cell proliferation via targeting the 3ʹ UTR of METTL3 mRNA^74^. Those studies suggested that METTL3 plays an oncogenic role in lung cancer.

### FTO plays an oncogenic role in acute myeloid leukemia

Acute myeloid leukemia (AML) is one of the most common type of hematopoietic malignancies with various genetic and molecular changes that shows different responses to treatment^75^. FTO was highly expressed in AMLs with MLL rearrangements and FLT3-ITD and/or NPM1 mutations^21^. Knockdown of FTO in MLL-rearranged AML inhibited cell growth. The comparison between overexpression of wild-type FTO and mutated FTO (H231A and D233A) in MLL-rearranged AML cells showed that only overexpression of wild-type FTO could promote cancer cell growth. Meanwhile, the m6A level was upregulated upon FTO knockdown, and was downregulated by overexpression of wild-type FTO rather than mutated FTO, indicating that FTO might regulate those phenotypes through modulating m6A modification. Similar results were obtained in AMLs with PML-RARA and FLT3-ITD/NPM1 mutations. Further in vivo experiments using bone marrow transplantation (BMT) assays showed that overexpression of FTO accelerated MLL-AF9-induced leukemogenesis. The mRNA transcripts of ankyrin repeat and SOCS box protein 2 (ASB2) and retinoic acid receptor-a (RARA) were confirmed to be significantly downregulated in accordance with hypo-methylated m6A peaks in FTO-overexpressing AML cells. ASB2 and RARA were two proteins found to be upregulated in all-trans-retinoic acid (ATRA)-induced differentiation of leukemia cells^76,77^. Further analysis confirmed that FTO inhibited ATRA-induced AML cell differentiation through regulating transcription of ASB2 and RARA. These data thus established a critical oncogenic role of FTO to promote leukemogenesis, and highlighted the contribution of FTO to ARTA-induced drug response in AML cells.

### Downregulation of m6A RNA methylation promotes glioblastoma

Glioblastoma is the deadliest primary brain tumor. The median survival time of glioblastoma patients is <15 months after diagnosis^78^. Glioblastoma stem cells (GSCs) are resistant to chemotherapy and radiotherapy, and promote the growth and invasion of cancer^79^. Differentiated GSC cell lines had an elevated m6A level while primary GSC cell lines exhibited lower m6A level^19^. Knockdown of METTL3 or METTL14 promoted the growth and self-renewal of GSCs, while overexpression of wild-type METTL3 rather than catalytically inactive METTL3 inhibited the growth and self-renewal of GSCs, indicating that METTL3 regulates GSCs’ self-renewal through its methyltransferase catalytic activity. Furthermore, knockdown of either METTL3 or METTL14 increased the growth of transplanted PBT003 cells in mice. MA2, an inhibitor of FTO, was shown to increase the m6A level and successfully reduced GSC-initiated tumor growth. These data shed lights on the m6A as a promising therapeutic target for glioblastoma and probably other cancers.

High ALKBH5 expression in GSCs was associated with a worse outcome^17^. Knockdown of ALKBH5 impaired the growth of GSCs, which can be rescued only by wild-type ALKBH5, but not the catalytic inactive mutant ALKBH5 H204A. After performing m6A sequencing and mRNA sequencing followed by ALKBH5 knockdown in GSCs, fork head box M1 (FOXM1) was found to be a candidate for ALKBH5-mediated GSCs growth. HUR increased its binding to pre-mRNA of FOXM1 because of the reduced m6A level, which resulted from ALKBH5 overexpression, thus increasing the stability of FOXM1 pre-mRNA^49^. Furthermore, the nuclear lncRNA FOXM1-AS was found to facilitate the interaction between ALKBH5 and FOXM1 nascent transcripts to promote the HUR binding. FOXM1-AS knockdown impaired the GSCs growth similar to ALKBH5 knockdown, and rescue of tumor growth of GSCs by FOXM1 overexpression after depletion of ALKBH5 or FOXM1-AS further proved the critical role of FOXM1 in GSC tumorigenesis.

### METTL14 inhibits hepatocellular carcinoma metastasis

m6A was reduced in hepatocellular carcinoma (HCC) tissue when compared with adjacent non-tumor or normal hepatic tissues^21^. After testing the mRNA expression of m6A-related factors between HCC and para-tumor tissue or normal tissue, METTL14 was found to be significantly lowered in HCC tissue. Downregulation of METTL14 showed a worse outcome in HCC patients and METTL14 mRNA expression was found to be further lower in metastatic tumors or portal vein tumor thrombus. METTL14 staining was negatively correlated with survival rates of HCC patients. Depletion of METTL14 revealed high metastatic capacity of HCC both in vitro and in vivo while overexpression of METTL14 suppressed tumor metastasis. As mentioned above, m6A promoted the miRNA processing by marking miRNA for recognition and processing by DGCR8^56^. Immunoprecipitation assay showed that METTL14 indeed coprecipitated with DGCR8. Different expressed miRNAs were selected between metastatic HCC and non-metastatic HCC. Downregulated miRNAs with m6A site in their pri-miRNAs in metastatic HCC might be targets of METTL14. As a result, miRNA 126 was found to be decreased while unprocessed pri-miR126 accumulated in METTL14-depleted cells. Consistently, forced expression of METTL14 resulted in an increased level of mature miR126 and pri-miR126 bound to DGCR8. Trans-well and invasion assay showed that METTL14 depletion-induced metastasis could be reversed by miR126 mimic while miR126 inhibitor increased the metastasis when METTL14 was forced overexpression, indicating that METTL14 suppressed the metastasis of HCC by increasing the miR126 level in an m6A-dependent manner.

miR145 is downregulated in various cancers including HCC^80–83^. YTHDF2 was highly expressed in HCC tissue while the expression level of miR145 was negatively correlated with YTHDF2^17^. Luciferase assay showed that miR145 directly targeted 3ʹ UTR of YTHDF2 mRNA. Overexpression of miRNA145 downregulated the mRNA and protein levels of YTHDF2 in HepG2 cells. YTHDF2 could recognize mRNA m6A site to mediate mRNA degradation and overexpression of YTHDF2 decreased the m6A level. Overexpression of miR145 increased the m6A levels of mRNAs and this could be blocked by YTHDF2 overexpression, while miR145 inhibitor decreased the m6A levels of mRNAs and rescued by siYTHDF2, suggesting that miRNA145 increased the m6A level through modulating YTHDF2.

### m6A facilitates DNA damage response secondary to radio- or chemotherapy

Targeted therapy based on inhibiting the DNA damage response in cancers offers the potential for a greater therapeutic window of radiotherapy or DNA-damaging chemotherapy^84^. Interestingly, m6A antibody stained DNA damage sites generated by UV laser micro-irradiation in U2OS bone osteosarcoma cells in a dose-dependent manner^18^. A time course experiment showed the response peaked at 2 min after irradiation and diminished over the following 8 min. RNase A treatment of cells removed m6A stains accumulation at damage sites, indicating that most of the signal is derived from polyadenylated RNA. METTL3 and METTL14 were stained in damage sites and knockdown of METTL3 decreased the m6A level in damage sites, which was rescued by overexpression of METTL3 with catalytic activity but not the mutated non-catalytic METTL3. Depletion of m6A demethylase FTO but not ALKBH5 increased the intensity of m6A RNA in damage sites. By using PARP inhibitors BYK, PJ-34, or olaparib, the m6A level was eliminated in U2OS cells, indicating that early DNA damage regulator PARP was required for the formation of m6A in response to UV. METTL3 knockout impaired the removal of cyclobutane pyrimidine dimers, delayed timely transcription re-initiation, increased the cell death, and decreased colony numbers in colony-formation assay after DNA damage. The overexpression of methylation catalytic METTL3 but not non-catalytic METTL3 rescued cell death in UV-treated U2OS cells with METTL3 knockout. DNA polymerases κ (Pol κ) localized to damage sites simultaneously with m6A RNA. And Pol κ overexpression rescued the defect in the removal of cyclobutane pyrimidine dimers associated with METTL3 loss, suggesting that Pol κ was a key effector of METTL3 in cyclobutane pyrimidine dimers repair. Therefore, PARPs, METTL3–METTL14 complex, FTO, and Pol κ formed a new DNA repair pathway in early UV-induced damage and m6A might be a promising target for combined therapy with radiotherapy or chemotherapy.

### Association between metabolism and m6A demethylase in cancer

FTO and ALKBH5 are α-ketoglutarate-dependent dioxygenases, which are competitively inhibited by the structurally related metabolite D-2-hydroxyglutarate (D2-HG). Isocitrate dehydrogenase 1 or 2 (IDH1/2) is frequently mutated in multiple types of human cancers such as glioblastoma^85^ and AML^86^. IDH1/2 catalyzes the NADP^+^-dependent oxidative decarboxylation of isocitrate into α-ketoglutarate, while mutant IDH1 and IDH2 lose their normal activity to produce α-ketoglutarate but gain a new activity to produce D2-HG, thus resulting in increased D2-HG and decreased α-ketoglutarate. Stable expression of IDH2 R140Q mutant and IDH2 R172K mutant in HEK293T cells resulted in a significantly higher m6A level and D2-HG level than wild-type IDH2^87^. The increase of D2-HG in R140Q- and R172K-expressing cells could be effectively inhibited by IDH2-mutant selective inhibitor AG-221, with global m6A levels downregulated to levels comparable with those of the isogenic IDH2-WT-expressing cells. Knockdown of FTO raised the m6A level in HEK293T expressed with IDH2 wild-type but not the mutated IDH2. Similar results were also obtained from IDH1/2-mutant and IDH1/2-WT AML cells that depletion of FTO only increased the m6A level in IDH1/2-WT AMLs but not IDH1/2-mutant AMLs. Therefore, IDH1/2 mutation increased m6A level by producing more D2-HG to competitively inhibit RNA demethylase FTO. Besides, other enzymes involved in D2-HG metabolism, such as 2-hydroxyglutarate dehydrogenase (2-HGDH), hydroxyacid-oxoacid transhydrogenase (HOT), and l-malate dehydroxygenase (l-malDH), might also influence FTO or ALKBH5 function and m6A level^88–91^. Furthermore, as FTO and ALKBH5 are both α-ketoglutarate-dependent, other metabolic pathways that produce α-ketoglutarate might also be involved in m6A regulation^92^. Besides, FTO and ALKBH5 also need employ ferrous iron as co-factor^32^. Iron is found to contribute to both tumor initiation and tumor growth. And cancer-related pathway, such as HIF and WNT pathways, may contribute to altered iron metabolism in cancer^93^. Iron metabolism and α-ketoglutarate metabolism in cancers need to be further addressed for their relationships with m6A.

SAM provides methyl for nearly all methylation reaction. MAT2A gene encodes the SAM synthetase that is expressed in all cells except liver cells^94^. SAM depletion leads to increased expression of MAT2A mRNA and overexpression of MAT2A promotes intron retention of MAT2A pre-mRNA^31^. METTL16 was found to methylate the fourth adenine of UACAGAGAA sequence of a hairpin in MAT2A 3ʹ UTR and promote the splicing of MAT2A pre-mRNA. In contrast, the F187G mutant METTL16, which did not bind MAT2A, had no effects on intron retention. MS2 tethering assay further revealed that METTL16 promotes splicing of MAT2A through vertebrate conserved regions (VCRs), but not methyltransferase activity domain. Notably, METTL16 was immunoprecipitated with MAT2A more efficiently after SAM depletion. Therefore, in Met-rich conditions (SAM is rich), METTL16 briefly occupied MAT2A due to enzyme turnover, and in Met-deprived conditions (SAM is limited), lack of methylation prolonged METTL16 occupation of MAT2A, which then drives splicing of MAT2A intron and produced more mRNA. In summary, m6A contributed to SAM homeostasis and on the other hand SAM homeostasis also regulated m6A modification.

## Inhibitors for m6A-related factors

Crystal structure of FTO^95^ and ALKBH5^96^ was determined, which would facilitate the understanding of substrate recognition and subsequent drug development. Linking 2OG derivatives with the substrate analogs has successfully developed selective inhibitors of histone demethylases containing a jumonji domain^97–99^. Similar strategy has applied to both FTO and ALKBH5^100^. However, these inhibitors are derivatives of 2OG, and therefore cellular 2OG might compete with them and weaken the inhibition. Rheinis the first potent FTO inhibitor^101^, which was found to inhibit FTO by competitively binding the catalytic domain against single-stranded RNA (ssRNA) substrate, and Rhein also effectively inhibited m6A demethylation in vitro and increased cellular levels of m6A. Despite the ability to inhibit FTO, the selectivity of Rhein was poor. Meclofenamic acid (MA) was found to be a more selective inhibitor of FTO, for it could bind and stabilize FTO but had minimal influence on ALKBH5. It was because the first loop in the FTO nucleotide recognition lid (NRL) provided hydrophobic interactions with MA, whereas ALKBH5 lacked this loop^96^. MA2 is the ethyl ester derivative of MA, which achieved better cell penetration. MA2 treatment increased the cellular levels of m6A and had no significant effects in cells with FTO depletion and ALKBH5 overexpression^102^. In addition to MA, other FTO inhibitors with special structural binding site of FTO were identified, such as *N*-(5-chloro-2,4-dihydroxyphenyl)-1-phenylcyclobutanecarboxamide (NCDPCB) and 4-chloro-6-(6′-chloro-7′-hydroxy-2′,4′,4′-trimethyl-chroman-2′-yl) benzene-1,3-diol (CHTB) (Table 3). However, it remains unknown whether those inhibitors influence ALKBH5.

**Table 3:** Inhibitors for m6A demethylases

Abbreviation of inhibitor	Target FTO?	Target ALKBH5?	Inhibit demethylase activity?	References (Pubmed ID)
IOX3	YES	YES	NO	24489119 25830347
N-CDPCB	YES	NA	YES	26314339
CHTB	YES	NA	YES	26915401
MA	YES	NO	YES	25452335
Citrate	YES	YES	NA	24778178
Rhein	YES	NA	YES	23547775

*m6A* N6-methyladenosine, *FTO* fat mass and obesity-associated, *ALKBH5* AlkB homologue 5, *IOX3* (1-chloro-4-hydroxyisoquinoline-3-carbonyl)glycine, *N-CDPCB* *N*-(5-chloro-2,4-dihydroxyphenyl)-1-phenylcyclobutanecarboxamide, *CHTB* 4-chloro-6-(6′-chloro-7′-hydroxy-2′,4′,4′-trimethyl-chroman-2′-yl)benzene-1,3-diol, *MA* meclofenamic acid, *NA* not available

IOX3 is an inhibitor of the HIF prolyl hydroxylases, which was found to bind non-covalently to the active site of FTO and decrease cellular protein expression of FTO^103,104^. However, this inhibitor failed to alter the m6A level inside of cells. IOX3 also could bind to ALKBH5 in a covalent attachment^96^. The citrate competed out 2OGs and Mn(ii) in the active site of ALKBH5 under the crystallization conditions^105^, which could be a modest inhibitor of ALKHB5. Though many kinds of inhibitors targeting m6A demethylases were identified, their effects were rarely validated in vivo. We expect to see more inhibitors targeting m6A-related factors both in vitro and in vivo.

## Conclusions and perspective

Being precisely regulated by various “writers”, “erasers”, and “readers”, m6A modification involves in almost every step in mRNA metabolism. In addition, it influences the processing of lncRNA and miRNA as well. m6A-modified RNAs experience a fast journal from RNA processing to degradation, and m6A controls cellular differentiation and pluripotency, both of which are associated with cancer progression. m6A plays important roles in metabolism, stem cell self-renewal, and metastasis in various cancers, indicating that m6A modification could be targeted for the prevention and treatment of human cancers. Indeed, FTO-specific inhibitor MA2 suppresses GSC-initiated tumor development in an m6A-dependent manner. However, more selective and powerful drugs targeting m6A-related factors are expected to be explored. The side effects of those inhibitors should also be considered, for m6A influences gene expression in many aspects. Moreover, the formation of m6A is affected by the level of methyl group from SAMs, and m6A demethylases FTO and ALKBH5 are Fe(ii) and α-ketoglutarate dependent. Therefore, the regulation of metabolism in cancer cells would have a profound impact on the dynamic regulation of m6A. Further studies are also needed to evaluate the biological relevance and diagnostic value of m6A in human cancers.
